# Circadian Control of Metabolism by the Clock Component TOC1

**DOI:** 10.3389/fpls.2021.683516

**Published:** 2021-06-14

**Authors:** Luis Cervela-Cardona, Takuya Yoshida, Youjun Zhang, Masaaki Okada, Alisdair Fernie, Paloma Mas

**Affiliations:** ^1^Centre for Research in Agricultural Genomics, CSIC-IRTA-UAB-UB, Barcelona, Spain; ^2^Max-Planck-Institut für Molekulare Pflanzenphysiologie, Potsdam, Germany; ^3^Center of Plant Systems Biology and Plant Biotechnology, Plovdiv, Bulgaria; ^4^Consejo Superior de Investigaciones Científicas, Barcelona, Spain

**Keywords:** circadian clock, TIMING OF CAB EXPRESSION1, metabolism, FUMARASE 2, *Arabidopsis thaliana*

## Abstract

Photosynthesis in chloroplasts during the day and mitochondrial respiration during the night execute nearly opposing reactions that are coordinated with the internal cellular status and the external conditions. Here, we describe a mechanism by which the Arabidopsis clock component TIMING OF CAB EXPRESSION1 (TOC1) contributes to the diurnal regulation of metabolism. Proper expression of TOC1 is important for sustaining cellular energy and for the diel and circadian oscillations of sugars, amino acids and tricarboxylic acid (TCA) cycle intermediates. TOC1 binds to the promoter of the TCA-related gene *FUMARASE 2* to repress its expression at night, which results in decreased fumarate accumulation in TOC1 over-expressing plants and increased in *toc1-2* mutant. Genetic interaction studies confirmed that over-expression of FUMARASE 2 in TOC1 over-expressing plants alleviates the molecular and physiological energy-deprivation phenotypes of TOC1 over-expressing plants. Thus, we propose that the tandem TOC1-FUMARASE 2 is one of the mechanisms that contribute to the regulation of plant metabolism during the day and night.

## Introduction

The circadian function is particularly relevant in plants, regulating nearly every aspect of growth, development and responses to internal and external cues (Greenham and McClung, 2015). The circadian molecular circuitry has been largely examined in the plant model system *Arabidopsis thaliana* (Sanchez and Kay, 2016). An essential component of the *Arabidopsis* circadian oscillator is the pseudo-response regulator known as TIMING OF CAB EXPRESSION1/PSEUDO RESPONSE REGULATOR1 (TOC1/PRR1) (Strayer et al., 2000; Makino et al., 2002). Rhythmic gene expression and protein accumulation of TOC1 is important in the control of circadian period by the clock (Strayer et al., 2000; Makino et al., 2002; Más et al.,2003a,b; Gendron et al., 2012; Huang et al., 2012; Pokhilko et al., 2012; Fung-Uceda et al., 2018). TOC1 exerts its function by repressing morning- and evening-expressed oscillator genes at the core of the clock (Gendron et al., 2012; Huang et al., 2012; Pokhilko et al., 2012). Many rhythmic outputs are affected by TOC1 miss-expression including among others, the cell cycle (Fung-Uceda et al., 2018), age-associated circadian period changes in leaves (Kim et al., 2016), growth (Más et al.,2003a,b; Yamashino et al., 2008), or flowering time (Somers et al., 1998; Más et al., 2003b; Niwa et al., 2007).

The circadian clock influences the timing of metabolism in animals, and thus, clock disruption results in metabolic disorders (Xu et al., 2008; Dibner and Schibler, 2015; Gill et al., 2015; Brown, 2016). In plants, metabolism relies in essence on two energy-related organelles: chloroplasts and mitochondria (Sweetlove and Fernie, 2013). Chloroplasts convert light energy into sugars, whereas mitochondria turn the nutrients into energy to cover for the cellular energetic demands (Millar et al., 2011). Photorespiration also supplies a large amount of NADH to mitochondria (Lim et al., 2020). Photosynthesis in chloroplasts and mitochondrial respiration are closely connected (Nunes-Nesi et al., 2011) but execute nearly opposing reactions. Thus, these activities must be precisely coordinated in sync with the internal cellular status and the external conditions. Fumarate is one of the intermediates of the TCA cycle (Nunes-Nesi et al., 2013) that accumulates to high amounts in *Arabidopsis* leaves due to the activity of two fumarase (fumarate hydratase) (FUM) enzymes (FUM1 and FUM2) (Chia et al., 2000; Araújo et al., 2011). *Arabidopsis* FUM2 activity accounts for the majority of the fumarate accumulation in leaves (Chia et al., 2000; Nunes-Nesi et al., 2007). *FUM2* expression and fumarate accumulation follow a diurnal oscillation (Chia et al., 2000; Nunes-Nesi et al., 2007; Pracharoenwattana et al., 2010), with higher accumulation during the light period. Fumarate and starch have been proposed as alternative carbon sinks for photosynthate (Chia et al., 2000; Tschoep et al., 2009; Pracharoenwattana et al., 2010).

The connection of the circadian clock with chloroplasts and photosynthetic activity has been firmly established (McClung et al., 2000; Dodd et al., 2005; Haydon et al., 2013; Noordally et al., 2013; Atkins and Dodd, 2014). Diel or circadian rhythms in metabolism-related gene expression or protein accumulation have been also reported (McClung et al., 2000; Michalecka et al., 2003; Blasing et al., 2005; Elhafez et al., 2006; Gibon et al., 2006; Lee et al., 2010; Flis et al., 2019). The use of single, double and triple clock mutant plants has shown altered gene expression and disrupted accumulation of organic acids and other metabolites (Fukushima et al., 2009; Sanchez-Villarreal et al., 2013; Flis et al., 2019) indicating that the circadian clock is important for proper metabolism. Here, we combine a wide-range of approaches including transcriptional, metabolomic, chromatin immunoprecipitation (ChIP) and genetic interaction studies to show that TOC1 function is important in the control of cellular metabolism at least in part through the oscillatory regulation of fumarase expression. We propose that TOC1 might function as a rheostat in the regulation of metabolism in sync with the day and night cycles.

## Materials and Methods

### Plant Material, Growing Conditions and Luminescence Assays

*Arabidopsis thaliana* lines (Columbia, Col-0) were surface sterilized dried on sterile filter paper in a laminar flow cabinet and sown on plates containing Murashige and Skoog (MS) agar medium supplemented or not (as specified for each experiment) with 3% (w/v) sucrose. Following 48 h of stratification at 4°C in the dark, plates were transferred to environmentally-controlled chambers (Inkoa Sistemas) and plants were grown under different photoperiodic conditions (Short Days, ShD, 8 h light:16 h dark; Long Days, LgD, 16 h light:8 h dark; LD, 12 h light:12 h dark) for 10–14 days with 60–100 μmol m^–2^s^–1^ of cool white fluorescent light at 22°C. For experiments under constant light conditions, plants synchronized under light/dark cycles were transferred to constant light (LL) for 2 days before samples were collected. The TOC1-ox (Huang et al., 2012), TOC1 RNAi (Más et al., 2003a), *toc1-2* (NASC, N2107710) and TMG-YFP/*toc1-2* (Huang et al., 2012) plants were described elsewhere. Plant transformation was performed by floral dipping protocols using *Agrobacterium tumefaciens* (GV2260)-mediated transfer (Clough and Bent, 1998). *In vivo* luminescence assays were performed as previously described (Takahashi et al., 2015). Briefly, 6 day-old seedlings synchronized under LD cycles were transferred to 96-well plates and released into LL conditions. Analyses were performed with a LB960 luminometer (Berthold Technologies) using the Microwin software (Mikrotek Laborsysteme).

### Plasmid Construction

The FUM2-ox construct was generated by PCR-mediated amplification of the *FUM2* coding sequence (CDS) followed by subcloning into the pENTR/D-TOPO vector (Invitrogen). The resulting vector containing the *FUM2* CDS was used to transform chemically competent *Escherichia coli* cells (one shot TOP10 cells, Gateway^®^). The *FUM2* CDS was introduced in the plant destination vector pGWB514 (35S pro, C-3xHA) (Nakagawa et al.,2007a,b) by homologous recombination using the LR reaction (Gateway^®^). Expression vectors (containing the *FUM2* CDS fused to 4x-MYC tag and expressed under the control of the 35S promoter) were also used to transform chemically competent *E. coli* cells (one shot TOP10, Gateway^®^). WT and TOC1-ox plants were transformed with the FUM2-ox construct to generate the single FUM2-ox and the double FUM2-ox/TOC1-ox plants, respectively.

### RNA Extraction and Reverse Transcription Quantitative PCR

About 20 seedlings of 12–14 days after stratification (das) were collected every 4 h over a diurnal or circadian cycle. RNA was purified using the Maxwell 16 LEV simply RNA Tissue kit (Promega). RNA was incubated with RNase-free TURBO DNase (Ambion) to eliminate genomic DNA contamination. Single strand cDNA was synthesized using 1 μg of RNA using iScript^TM^ Reverse Transcription Supermix for RT-Q-PCR (Bio-rad) or AffinityScript Q-PCR cDNA Synthesis Kit (Agilent). For qPCR analysis, cDNAs were diluted fivefold with nuclease-free water and qPCR was performed with 10% of diluted cDNA with Brilliant III Ultra-Fast SYBR Green qPCR Master Mix (Agilent) or iTag Universal SYBR Green Supermix (Bio-Rad) in a 96-well CFX96 Touch Real-Time PCR detection system (Bio-Rad). The ISOPENTENYL PYROPHOSPHATE:DIMETHYLALLYL PYROPHOSPHATE ISOMERASE 2 (*IPP2*) gene was used as control (Huang et al., 2012). Data were analyzed using the second derivative maximum method. Resulting Cp values were converted into relative expression values using the comparative Ct method (Livak and Schmittgen, 2001).

### Adenylate Measurements by HPLC

The ADP and ATP content was measured by High Performance Liquid Chromatography (HPLC) with 20 mg of fresh weight plant materials (Zhang et al., 2020). Briefly, 0.2 ml of 0.1 M HCL was mixed with the plant materials on ice. 15 μl extract/standard (different concentration) was mixed with 77 μl CP Buffer [62 mM, citric acid monohydrate and 76 mM (Na)_2_HPO_4_ × 2H_2_O] and 8 μl 45% chloroacetaldehyde and incubated at 80°C for 10 min. The mixture was then centrifuged at 16,000×*g* at 20°C for 30 min. 90 μl of the supernatant was then measured by the HPLC [Hyperclone C18 (ODS) column (Phenomenex)]. The result was calculated using the standard ADP and ATP gradient.

### Chromatin Immunoprecipitation Assays

Chromatin Immunoprecipitation assays were performed essentially as previously described (Perales and Más, 2007). Approximately 1 g of 12–14 das seedlings were sampled at Zeitgeber Time 7 (ZT7) and Circadian Time 7 (CT7 for TOC1-ox, and every 4 h for TMG plants. Samples were fixed in 30 ml of ice-cold fixation buffer (0.4 M Sucrose, 10 mM *Tris*-HCl pH 8.0, 1 mM EDTA, 1 mM PMSF, 1% Formaldehyde, 0.05% Triton X-100) for 15 min under vacuum. Fixation was stopped by addition of ice-cold glycine 0.125 M and vacuum incubation for 5 min. Seedlings were then washed three times with ice-cold water and dried. The resulting seedlings were grounded in liquid nitrogen and the powder was filtered twice with miracloth. Extraction was performed with extraction buffer I (0.4 M Sucrose, 10 mM *Tris*-HCl pH 8.0, 5 mM β-mercaptoethanol, 1 mM PMSF, 5 μg/ml Leupeptin, 1 μg/ml Aprotinin, 5 μg/ml Antipain, 1 μg/ml Pepstatin, 5 μg/ml Chymostatin, and 50 μM MG132). Nuclei were then purified by centrifugation at 4°C for 20 min at 1,000*g*. Nuclei were washed four times by centrifugation at 4°C for 20 min at 1,000*g* with 2 ml of extraction buffer II (0.25 M Sucrose, 10 mM *Tris*-HCl pH 8.0, 10 mM MgCl2, 1% Triton X-100, 5 mM β-mercaptoethanol, 1 mM PMSF, 5 μg/ml Leupeptin, 1 μg/ml Aprotinin, 5 μg/ml Antipain, 1 μg/ml Pepstatin, 5 μg/ml Chymostatin, and 50 μM MG132). Nuclei were resuspended in 1 ml of nuclei lysis buffer (50 mM *Tris*-HCl pH 8.0, 10 mM EDTA, 1% SDS, 5 μg/ml Leupeptin, 1 μg/ml Aprotinin, 5 μg/ml Antipain, 1 μg/ml Pepstatin, 5 μg/ml Chymostatin, and 50 μM MG132). 300 μl of chromatin was sonicated to approximately 500–1,000 bp fragments with a sonicator (Bioruptor Next Generation, Diagenode). Following centrifugation at 12,000×*g* for 10 min at 4°C, 100 μl of soluble chromatin (the supernatant) was diluted in 400 μl of ChIP dilution buffer (15 mM *Tris*-HCl pH 8.0, 150 mM NaCl, 1% Triton-X-100, 1 mM EDT, 1 mM PMSF, 5 μg/ml Leupeptin, 1 μg/ml Aprotinin, 5 μg/ml Antipain, 1 μg/ml Pepstatin, 5 μg/ml Chymostatin, and 50 μM MG132) and incubated overnight at 4°C with 50μl of Magnetic beads (Dynabeads protein G, Invitrogen) and with 1:1,000 (−) of Anti-MYC antibody (Sigma-Aldrich) for assays with TOC1-ox plants or Anti-GFP (Invitrogen by Thermo Fisher Scientific) antibody for the assays with TMG plants (Invitrogen). Immunocomplexes were washed twice with 900 μl of low salt buffer (20 mM *Tris*-HCl pH 8.0, 150 mM NaCl, 1% Triton X-100, 0.1% SDS, 2 mM EDTA), twice with 900μl of high salt buffer (20 mM Tris-HCl pH 8.0, 500 mM NaCl, 1% Triton X-100, 0.1% SDS, 2 mM EDTA), twice with 900 μl of LiCl wash buffer (10 mM *Tris*-HCl pH 8.0, 0.25 M LiCl, 1% NP-40, 1% Sodium Deoxycholate, 1 mM EDT) and twice with 900 μl of TE buffer (10 mM *Tris*-HCl pH 8.0, 1 mM EDT). Immunocomplexes were eluted 300 μl with 1% SDS and 0.1 M NaHCO3 followed by 1 h at 65°C to break the bonds between the antibodies and the proteins. Next, 220 mM NaCl were added to precipitate the DNA, following incubation overnight at 65°C for reverse cross-linking. Immunoprecipitated DNA was isolated using the QIAquick kit (Qiagen) following the manufacturer instructions. ChIPs were quantified by QPCR analysis using a 96-well CFX96 Touch Real-Time PCR Detection System (BioRad). Crossing point (Cp) calculation was used for quantification using the Absolute Quantification analysis by the 2nd Derivative Maximum method. ChIP values for each set of primers were normalized to Input values.

### GC-MS Based Metabolite Profiling

Metabolite analyses were performed essentially as previously described (Lisec et al., 2006). Briefly, seedlings were collected at the indicated time points, immediately frozen in liquid nitrogen, and stored at −80°C until further analysis. Metabolite extraction was performed by rapid grinding in liquid nitrogen and immediate addition of the extraction buffer (1,400 μL of methanol plus 60 μL of 0.2 mg ribitol mL^–1^ water). The extraction, derivatization, standard addition, and sample injection were conducted as described (Lisec et al., 2006) with minor modifications in the equipment. An autosampler Gerstel Multi-Purpose system (Gerstel GmbH & Co., KG, Mülheim an der Ruhr, Germany) was used to inject the samples to a chromatograph coupled to a time-of-flight mass spectrometer (GC-MS) system, Leco Pegasus HT TOF-MS (LECO Corporation, St. Joseph, MI, United States). Metabolites were identified by comparison with database entries of authentic standards (Kopka et al., 2005; Schauer et al., 2005). Chromatograms and mass spectra were evaluated using the Chroma TOF 1.0 (Leco^1^) and the TagFinder 4.0 software (Luedemann et al., 2012). The relative content of metabolites was calculated by normalization of signal intensity to that of ribitol, which was added as an internal standard, and by the fresh weight of the material. All data were also processed using the Xcalibur 4.0 software (Thermo Fisher Scientific, Waltham, MA, United States) to verify the metabolite identification and annotation. Identification and annotation of detected peaks followed the recommendations for reporting metabolite data (Fernie et al., 2011).

### Rosette Phenotyping

Whole rosettes of 35-day old seedlings growing on soil pots were detached and immediately weigh in a precision balance (OHAUS Pioneer PA114C Analytical Balance) to calculate fresh weight. Detached rosettes were immediately placed on top of a transilluminator and photographed (NIKON D7000) for further analysis. Rosette images were processed with ImageJ software (Schneider et al., 2012). First, images were split using the RGB color tool. Blue channel images were then converted to 8 bit black/white images. After the same scale was set for all samples, rosettes were defined using the wand tracing tool and the area and the perimeter was obtained.

## Results

### Circadian Regulation of Nuclear-Encoded Mitochondrial-Related Transcriptome

**FIGURE 1:**
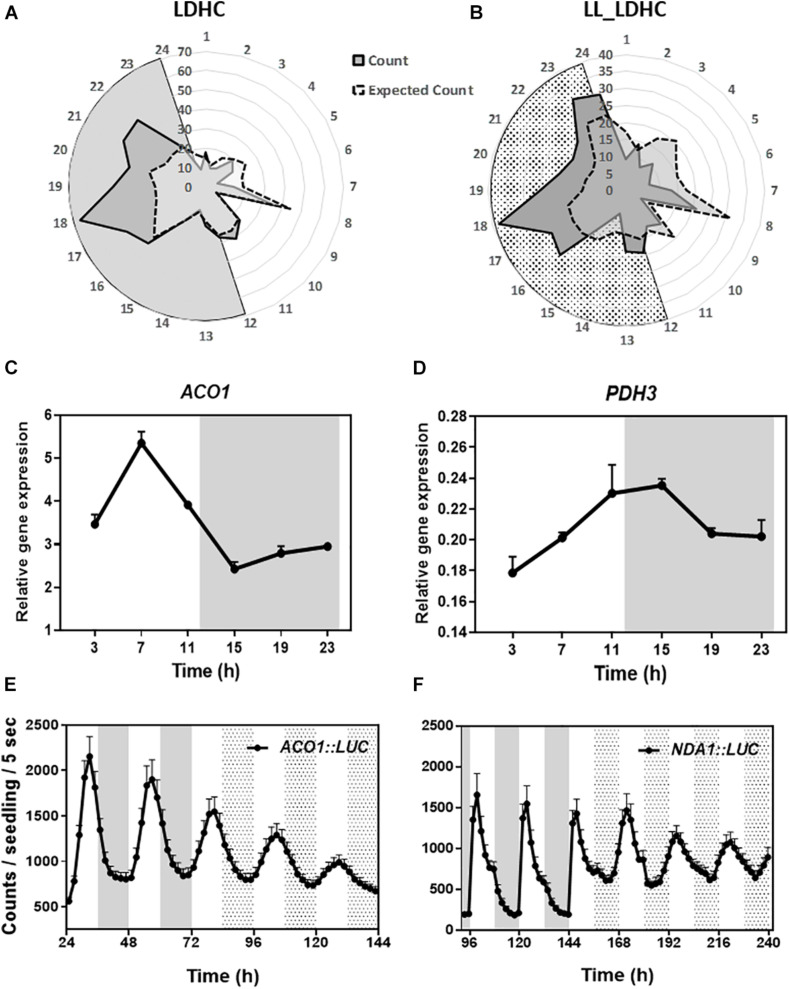
Diel and circadian oscillation of mitochondrial-related gene expression. Peak phases of expression of mitochondrial-related genes represented in radial plots under **(A)** LDHC and **(B)** LL_LDHC conditions. Radial axis: 24-dawn, or subjective dawn; 12-dusk, or subjective dusk. Time course analyses by RT-QPCR of **(C)** *ACONITASE 1* (*ACO1*) and **(D)** *MITOCHONDRIAL PYRUVATE DEHYDROGENASE SUBUNIT 2-1* (*PDH3*) gene expression under diurnal conditions. Data are represented as the mean + SEM. Promoter activities of **(E)** *ACO1* fused to *LUCIFERASE* (*ACO1:LUC*) and **(F)** *ALTERNATIVE NAD*(*P*)*H DEHYDROGENASE 1* (*NDA1*) fused to *LUC* (*NDA1:LUC*) under diurnal and free-running conditions. Data are represented as the mean + SEM. The white and gray areas in panels **(A,C–F)** represent day and night, respectively. The white and dotted areas in panels **(B,E,F)** represent subjective day and subjective night, respectively. Three biological replicates were performed for all experiments, with plants grown independently, and with samples collected, processed and analyzed at different times.

To determine the oscillatory transcription of nuclear-encoded mitochondrial-related genes, we used the web tool DIURNAL (Mockler et al., 2007; Michael et al., 2008) and found that about 65% of the mitochondrial-related genes (Chrobok et al., 2016) displayed an oscillatory pattern of expression under diel conditions with spread peak phases but particularly enriched in the middle of the night at ZT17-22 (Figure 1A). Time course assays by Reverse Transcription-Quantitative Polymerase Chain Reaction (RT-QPCR) confirmed the diurnal oscillation of selected genes belonging to mitochondrial-related metabolic pathways (Figures 1C,D and Supplementary Figures 1A–J). To ascertain whether the oscillatory pattern of expression is controlled by the circadian clock, we also used the web tool DIURNAL (Mockler et al., 2007; Michael et al., 2008) and performed the analyses under LL conditions. Our studies revealed that around 42% of the mitochondrial-related genes showed a circadian oscillation (Figure 1B). These results were also confirmed by RT-QPCR analyses (Supplementary Figures 1K–T). Furthermore, analyses of *ACONITASE1* (*ACO1*) and *ALTERNATIVE NAD(P)H DEHYDROGENASE 1* (*NDA1*) promoters fused to the *LUCIFERASE* also uncovered the rhythmic promoter activities under both diel and circadian conditions (Figures 1E,F). Altogether, the data from the web tool DIURNAL, our RT-QPCR and promoter activity analyses, all indicate the diel and circadian oscillation of some nuclear-encoded mitochondrial-related genes.

### TOC1 Regulates the Circadian Expression of Mitochondrial-Related Genes

**FIGURE 2:**
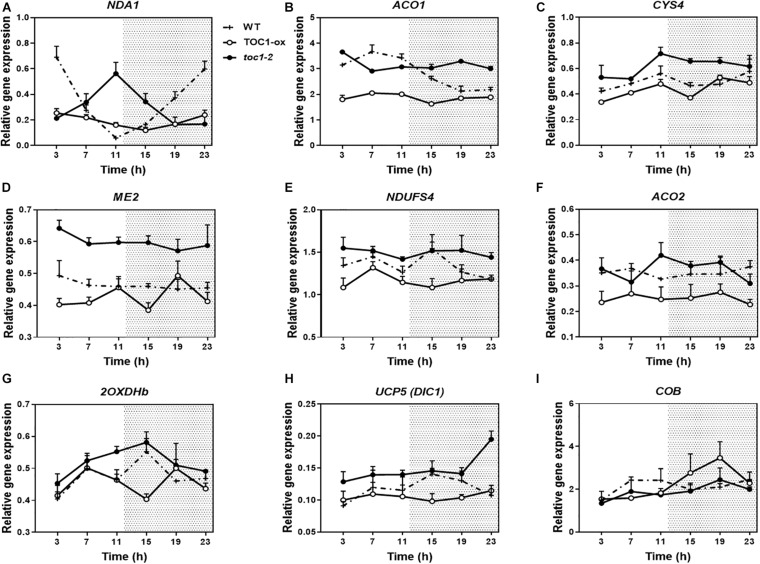
Miss-expression of mitochondrial-related genes in TOC1-ox and *toc1-2* mutant plants. Time course analyses of mitochondrial-related gene expression in WT, TOC1-ox and *toc1-2* plants over a circadian cycle. Plants were entrained under LD cycles and transfer for 2 days under LL conditions. Expression of **(A)** *NDA1*, **(B)** *ACO1*, **(C)** *CITRATE SYNTHASE 4* (*CYS4)*, **(D)** *NAD-DEPENDENT MALIC ENZYME 2* (*ME2*), **(E)** *NADH:UBIQUINONE OXIDOREDUCTASE FE-S PROTEIN4 (NDUFS4)*, **(F)** *ACONITASE 2* (*ACO2)*, **(G)** *2-OXOGLUTARATE DEHYDROGENASE E1-2* (*2OXDHb)*, **(H)** *DICARBOXYLATE CARRIER 1* (*UCP5/DIC1)* and **(I)** *CYTOCHROME B (COB)*. Relative expression was obtained by RT-Q-PCR analyses. Data are represented as the mean + SEM. The white and dotted areas in represent subjective day and subjective night, respectively. Three biological replicates were performed for all experiments, with plants grown independently, and with samples collected, processed and analyzed at different times.

Previous transcriptomic profiles showed that the clock components PRR9, 7, and 5 might be important in the control of metabolism (Fukushima et al., 2009). The study was conducted with plants growing in medium containing sucrose and excluded the founder member of the PRR family, TOC1, which plays distinct roles within the circadian system and in the control of particular outputs. Our analyses using TOC1 miss-expressing plants showed that the rhythmic expression of genes encoding proteins belonging to the mitochondrial electron transport chain (ETC), the tricarboxylic acid (TCA) cycle, as well as expression of NADH dehydrogenases, alternative oxidases (AOX) and mitochondrial transporters (TRANSP) was affected in TOC1 over-expressing and mutant plants grown in a medium without sucrose (Supplementary Figures 2A–C). Peak accumulation appeared to be delayed in TOC1-ox and advanced in *toc1-2*. Comparative analyses of individual genes in the three genotypes confirmed the altered expression, showing that some genes were down-regulated in TOC1-ox and conversely, up-regulated in *toc1-2* (Figures 2A–I). Less frequently, the amplitude of some genes was increased in TOC1-ox (Figure 2I). As TOC1 functions as a general repressor (Gendron et al., 2012; Huang et al., 2012; Pokhilko et al., 2012), these genes might be indirectly regulated by the miss-expression of TOC1.

### TOC1 Regulates the Diurnal and Circadian Accumulation of Central Metabolites and Cellular Energy

**FIGURE 3:**
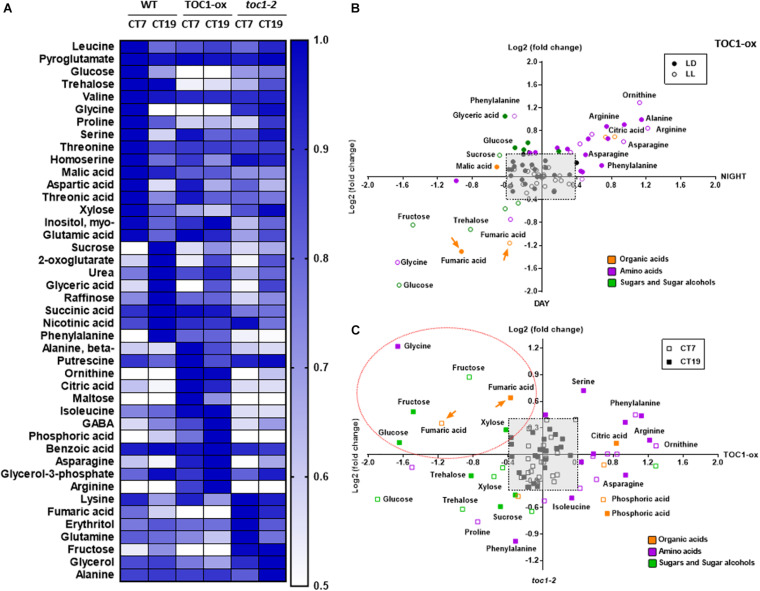
TIMING OF CAB EXPRESSION1 regulates the diurnal and circadian accumulation of metabolites. **(A)** Heat map of GC-MS-analyzed metabolites in WT, TOC1-ox and *toc1-2* plants. Plants were entrained under 12 h light: 12 h dark cycles and then transferred for 2 days to LL before sampling at CT7 and CT19. **(B)** Dispersion plots of metabolite fold change (day versus night) under LD (12 h light: 12 h dark cycles) and LL (subjective day versus subjective night) in TOC1-ox relative to WT. **(C)** Dispersion plots of metabolite fold change (*toc1-2* versus TOC1-ox) under LL. Metabolites were clustered per class into organic acids (orange), amino acids (purple), and sugars and sugar alcohols (green). Relative metabolite content was normalized to the mean of WT plants and fold-change values were log2 transformed. Log2 fold differences < [0.4] were not considered (pale gray square). Orange dotted oval denotes metabolites down-regulated in TOC1-ox and up-regulated in *toc1-2*. Data are the result of 3–5 biological replicates, with plants grown independently at different times.

To examine metabolite accumulation in Wild-Type (WT) and TOC1 miss-expressing plants, we performed targeted metabolomics by Gas Chromatography/Mass Spectrometry (GC/MS) analyses in plants grown in a medium without sucrose (Supplementary Table 1). In WT plants, a number of metabolites displayed diurnal fluctuations with higher accumulation during the day at ZT7 than at night at ZT19 (Supplementary Figure 3A). Examples include sugars such as glucose, amino acids such as serine, or TCA intermediates such as succinic acid. The oscillatory pattern was altered in TOC1-ox for a subset of these metabolites, with increased accumulation either in the morning or in the evening (Supplementary Figure 3A and Figure 3B). Metabolites with high amplitude fluctuations in WT showed decreased accumulation in TOC1-ox (Figure 3B and Supplementary Figure 3A) and were slightly increased in *toc1-2* (Supplementary Figures 3A,B). Under LL conditions, metabolites also fluctuated with increased accumulation during the subjective day (e.g., glycine) or night (e.g., phenylalanine) (Figure 3A). The rhythmic fluctuations were altered in TOC1-ox and *toc1-2* mutant plants (Figures 3A–C and Supplementary Figure 3B). Amino acids accumulated either during the day or night in TOC1-ox, whereas sugars and fumaric acid displayed a clear reduction. Conversely, fructose, fumaric acid or the amino acid lysine were increased in *toc1-2* (Supplementary Figures 3A,B). Thus, proper expression of TOC1 is important for the diel and circadian oscillations of sugars, amino acids and TCA intermediates.

**FIGURE 4:**
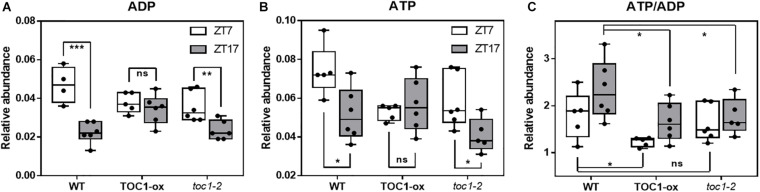
Proper expression of TOC1 is important for the regulation of energy homeostasis. HPLC analyses of **(A)** ADP, **(B)** ATP and **(C)** ATP/ADP ratio in WT, TOC1-ox and *toc1-2* plants that were entrained under 12 h light: 12 h dark cycles and sampled at ZT7 and ZT17. Two-tailed, *T*-test *p*-values: *p < 0.05; **p < 0.01; ***p < 0.001. ns, not significant. Data are the result of five biological replicates, with plants grown independently at different times.

We next examined whether a main output of metabolism, i.e., energy production in the form of ATP, was altered when the clock is not properly functioning. We used a highly sensitive method to quantify total adenine nucleotides (Zhang et al., 2020). Our results showed a clear variation in adenosine diphosphate (ADP) and adenosine triphosphate (ATP) content during the day compared to the night in WT plants (Figures 4A,B). The amplitude of the oscillation was considerably reduced in TOC1 miss-expressing plants suggesting that proper *TOC1* expression is necessary for sustaining diurnal energy homeostasis (Figures 4A,B). Calculation of the ATP/ADP ratio, a measure of the energetic homeostasis, showed that the day-night differences in WT plants were reduced or lost in TOC1 miss-expressing plants (Figure 4C). Consistent with the constitutive over-expression, the ATP/ADP ratio was reduced in TOC1-ox at both time points, whereas the effect of *toc1-2* mutation was more evident during the night (Figure 4C). The results connect the circadian function of TOC1 with metabolism and cellular energy.

Our results and those previously reported (Fukushima et al., 2009) suggest a role for the PRR protein family modulating the rhythms of primary metabolites. However, it is unknown whether this connection is direct or occurs through other molecular clock components and/or clock outputs. As TOC1 functions as a general repressor (Gendron et al., 2012; Huang et al., 2012; Pokhilko et al., 2012), we compared metabolites miss-regulated in TOC1-ox and *toc1-2* relative to WT, and focused on those down-regulated in TOC1-ox and up-regulated in *toc1-2*. We reasoned that metabolites in this cluster might provide an indication of the possible direct regulation by TOC1 of genes involved in the generation of these metabolites. Our analyses uncovered the sugar fructose, the amino acid glycine, and the TCA intermediate fumaric acid as down-regulated in TOC1-ox and up-regulated in *toc1-2* under both LD (Supplementary Figure 3C, dotted orange oval) and LL (Figure 3C, dotted orange oval).

### TOC1 Controls the Diurnal and Circadian Expression of *FUM2* and Directly Binds to Its Promoter

**FIGURE 5:**
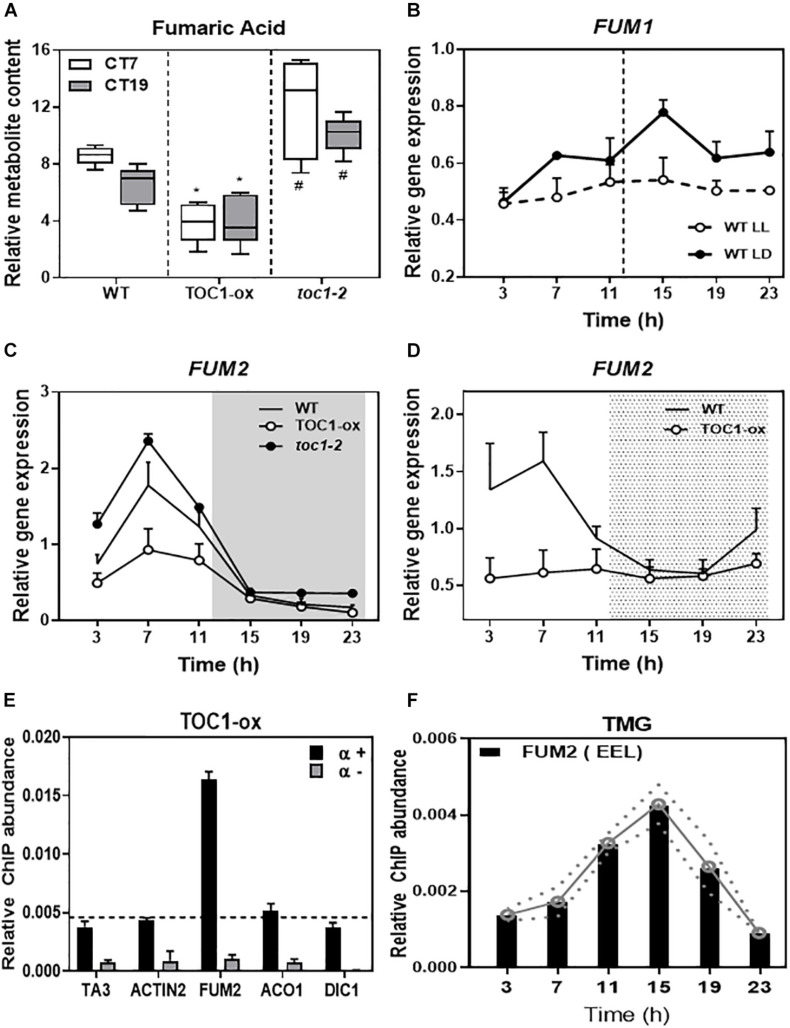
TIMING OF CAB EXPRESSION1 controls the diurnal and circadian expression of *FUM2* by direct binding to its promoter. **(A)** Relative fumaric acid content in WT, TOC1-ox and *toc1-2* plants entrained under LD cycles and then transferred for 2 days to LL before sampling at Circadian Time 7 (7 h after subjective dawn) CT7 and CT19. **(B)** Time course analyses of *FUM1* expression in WT plants grown under LD or transferred for 2 days to LL before sampling. **(C)** Time course analyses of *FUM2* expression in WT, TOC1-ox and *toc1-2* plants grown under LD. **(D)** Time course analyses of *FUM2* expression in WT and TOC1-ox plants grown under LD transferred for 2 days to LL before sampling. **(E)** Chromatin immunoprecipitation (ChIP) assays with TOC1-ox plants grown under LD and sampled at ZT7. ChIP enrichment was calculated relative to the input. Samples were incubated with an anti-MYC antibody (+α) or without antibody (-α). **(F)** ChIP assays with TMG plants grown under LD and sampled every 4 h over the diurnal cycle. ChIPs were performed with an anti-GFP antibody to immunoprecipitate the GFP-tagged TOC1 protein. ChIP enrichment was calculated relative to the input. The white and gray areas in panel **(C)** represent day and night, respectively. The white and dotted areas in panel **(D)** represent subjective day and subjective night, respectively. Three biological replicates were performed for all experiments, with plants grown independently, and with samples collected, processed and analyzed at different times. Two-tailed, *t*-test of WT Vs. TOX1ox *p*-values: *p < 0.05 and WT Vs. toc1-2 *p*-values: #< 0.05.

Fumaric acid is an intermediary of the TCA cycle synthesized by the activity of two fumarases (FUM1 and FUM2). As the diurnal and circadian oscillation of fumaric acid dampened low in TOC1-ox and high in *toc1-2* (Figure 5A and Supplementary Figure 4A), we focused on the possible regulation of the *FUM* genes by TOC1. Time course analyses showed that *FUM1* expression followed a low amplitude oscillation (Figure 5B) that was not manifestly altered in TOC1-ox (Supplementary Figure 4B). In contrast, *FUM2* expression clearly oscillated with a peak of expression in the middle of the day under different photoperiods and under LL conditions (Supplementary Figures 4C,D). The amplitude of *FUM2* expression was reduced in TOC1-ox and increased in *toc1-2* under entraining conditions (Figure 5C). Under LL, *FUM2* circadian peak of expression was abolished in TOC1-ox (Figure 5D) and up-regulated during the subjective day in *toc1-2* (Supplementary Figure 4D). These results suggest that the transcriptional effects of TOC1-ox and *toc1-2* correlate with the phenotypes of fumaric acid accumulation.

Our previous chromatin immunoprecipitation massive sequencing (ChIP-Seq) analyses have shown that *FUM2* is one of the targets of TOC1 (Huang et al., 2012). To examine whether regulation of *FUM2* gene expression by TOC1 occurs through direct binding to the *FUM2* locus, we performed ChIP assays followed by Quantitative PCR (QPCR). Our assays using TOC1-ox plants showed amplification of the *FUM2* promoter to a degree similar to that observed for a positive control (*CCA1* promoter) (Figure 5E and Supplementary Figure 4E). The promoters of other metabolism-related genes (*ACO1*, *DIC1*, *NDA1*, and *PDK*), a negative control (*ACTIN*) or samples processed without antibody (-α) did not show significant enrichment under our experimental conditions (Figure 5E and Supplementary Figure 4E). We also performed ChIP assays with TOC1 Minigen (TMG) seedlings expressing the *TOC1* genomic fragment under *TOC1* promoter (Más et al., 2003a). Our analyses revealed a rhythmic binding with peak enrichment around ZT15 (Figure 5F). Binding was observed in a *FUM2* promoter region containing circadian-related motifs including the Evening Element-like motif (EEL and AATATCT), previously identified as a TOC1-binding motif, CCA1 Binding Site (CBS and AAAAATCT) and Morning Element (ME and CCACAC) (Michael et al., 2008) (Figure 5E). Together, the results are consistent with a direct binding of TOC1 to the *FUM2* promoter to regulate its diurnal and circadian transcriptional expression that ultimately also correlates with fumaric acid abundance.

### Over-Expression of FUM2 Restores the Metabolism-Related Phenotypes of TOC1-ox

**FIGURE 6:**
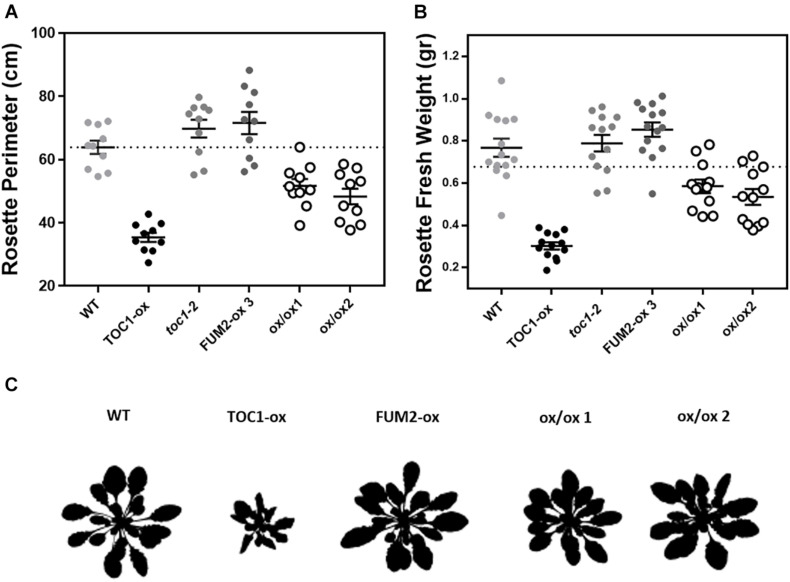
Over-expression of FUM2 partially restores the molecular and physiological phenotypes of TOC1-ox. Rosette **(A)** perimeter and **(B)** fresh weight of the indicated genotypes grown under long day (LgD) conditions. Every dot represents a single rosette. Dotted lines show the mean of WT. **(C)** Rosette 8 bits images. Data are represented as the mean + SEM. Three biological replicates were performed for all experiments, with plants grown independently, with samples collected, processed and analyzed at different times.

If the phenotypes observed in TOC1-ox are due to the repression of *FUM2*, its over-expression in the TOC1-ox background should decrease the severity of the phenotypes. Thus, we next conducted genetic interaction studies by transforming TOC1-ox plants with a FUM2 over-expressing construct (FUM2-ox) (Supplementary Figures 5A,B). The severity of the rosette size and fresh weight phenotypes of TOC1-ox was also significantly improved in the double FUM2/TOC1-ox plants compared to single TOC1-ox (Figures 6A,B and Supplementary Figure 5C). A similar phenotypic recovery was observed in two different double overexpressing lines (Figure 6C and Supplementary Figure 5C). These results suggest that the reduced expression of *FUM2* in TOC1-ox plants contributes to the observed phenotypes and that over-expression of FUM2 alleviates these phenotypes. The results also limit the possibility that the observed phenotypes of TOC1-ox plants are due to indirect pleiotropic effects.

**FIGURE 7:**
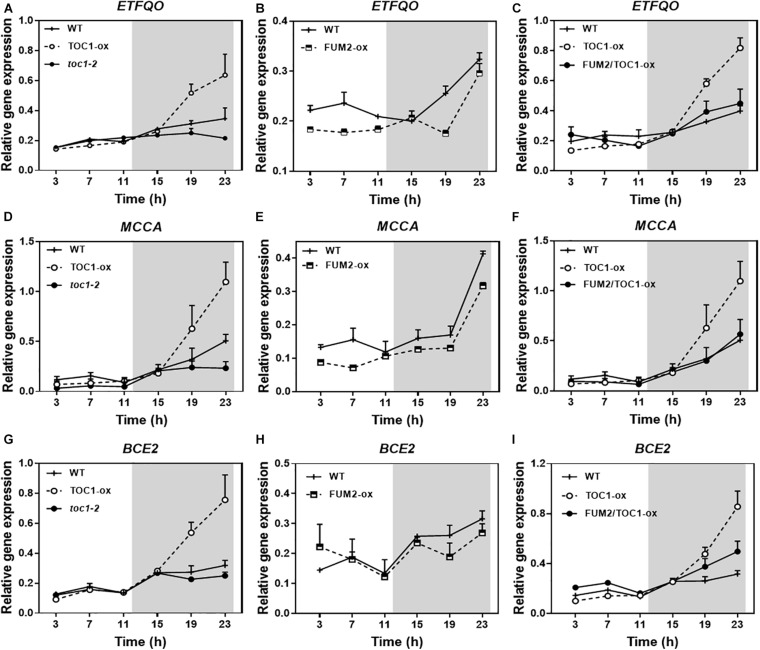
Genetic interaction studies of the molecular signatures showing energy deprivation. Time course analysis by RT-QPCR of **(A–C)** *ELECTRON-TRANSFER FLAVOPROTEIN: UBIQUINONE OXIDO-REDUCTASE* (*ETFQO*), **(D–F)** *METHYLCROTONYL-COA CARBOXYLASE* (*MCCA*) and **(G–I)** *DARK INDUCIBLE 3* (*BCE2/DIN3*) gene expression in the indicated genotypes grown under LD cycles. Data are represented as the mean + SEM. The white and gray areas represent day and night, respectively. Some data are repeated in different graphs to facilitate the comparisons among genotypes. Two (FUM2-ox) and three (WT, TOC1-ox, *toc1-2*, and FUM2/TOC1-ox) biological replicates were performed, with plants grown independently, with samples collected, processed and analyzed at different times.

Growing conditions that mimic energy deprivation trigger alternative metabolic pathways including the induction of genes involved in the catabolism of Branched-Chain Amino Acids (BCAA) (Pedrotti et al., 2018). Notably, the expression of a number of amino acid catabolic genes was up-regulated in TOC1-ox during the night (Figures 7A,D,G). The up-regulation was more evident under shorter photoperiods (Supplementary Figure 5A). The genes were up-regulated in TOC1-ox although the plants were grown under normal cycles and not under energy deprivation conditions in which these genes are induced. Other genes encoding amino acid catabolic enzymes as well as sugar starvation markers were also induced in TOC1-ox plants (Supplementary Figures 6B,C). Conversely, gene expression was down-regulated in FUM2-ox and in *toc1-2* (Figures 7A,B,D,E,H). The up-regulation of *BCCA* catabolic gene expression in TOC1-ox was overcome in the double FUM2/TOC1-ox plants (Figures 7C,F,I), suggesting that the observed phenotypes in TOC1-ox are indeed mediated by the reduced expression of *FUM2*. The results support a redirected proteolytic metabolism due to the altered energy status of TOC1 miss-expressing plants.

## Discussion

Temporal compartmentalization of metabolism is pervasive in unicellular and multicellular organisms. Due to their sessile nature and highly compartmentalized cells (Sweetlove and Fernie, 2013), an accurate spatiotemporal distribution of metabolic processes is particularly essential for plants. Clock-dependent mechanisms in cooperation with specific regulatory pathways allow plants to actively anticipate and prepare reactions in sync with internal metabolic signals and external environmental cues (Smith and Stitt, 2007; Sanchez-Villarreal et al., 2013). We have identified a molecular mechanism that connects the circadian clock with the regulation of metabolism in *Arabidopsis thaliana*.

Our results show that proper expression of TOC1 is important for the diel and circadian oscillations of sugars, amino acids and TCA intermediates. TCA intermediates, including fumarate were also found to accumulate in the triple *d975* (*PRR9, PRR7*, and *PRR5*) mutant plants (Fukushima et al., 2009). Compared to TOC1-ox, the phenotypes of *toc1-2* plants were less severe, suggesting a possible functional redundancy with other members of the PRR family (for instance PRR5). Overall, the metabolite profiles were comparable to those previously reported (Flis et al., 2019) with some differences that might be explained by the different plant developmental stages and growing conditions. Our results show that TOC1 regulates the expression of *FUM2*. Consistently, *FUM2* expression was down-regulated by induction of *REVEILLE8* (Hsu et al., 2013), a previously described activator of *TOC1* (Hsu et al., 2013; Xing et al., 2015; Ma et al., 2018). The altered fumarate accumulation in TOC1 miss-expressing plants suggests that the transcriptional regulation of *FUM2* is responsible for the observed changing patterns of fumarate. A similar conclusion can be drawn by our genetic interaction studies using FUM2-ox plants. As fumarate and starch serve as alternative carbon sinks for photosynthate (Chia et al., 2000; Tschoep et al., 2009; Pracharoenwattana et al., 2010), the regulation of fumarate accumulation (and other metabolites) by TOC1 provides a novel way for the circadian clock to control cellular metabolism in addition to the one previously described through the control of starch metabolism.

We also found that TOC1 directly binds to the *FUM2* promoter to regulate its diurnal and circadian transcriptional expression. Although previous studies have provided evidence that multiple circadian clock outputs regulate diel patterns of C and N metabolism, the binding of TOC1 to the *FUM2* promoter and the regulation of *FUM2* expression provide a novel direct molecular mechanism connecting TOC1 with circadian metabolism. Our results also suggest that under normal LD cycles, the energy demand in TOC1 miss-expressing plants is not properly supplied so that alternative pathways for generating energy are triggered. Many of the enzymes of the BCAA pathway are located in mitochondria (Taylor et al., 2004) and the BCAA genes display rhythms during the diurnal cycle (Blasing et al., 2005; Lee et al., 2010), being transcriptionally repressed by light (Ishizaki et al., 2005, 2006). It is plausible that the disruption of energy homeostasis in TOC1-ox triggers an alarm response to obtain energy from other sources, in this case the catabolism of branched amino acids. Analyses of *Arabidopsis tic* mutant plants showed an altered starch, carbohydrate, amino acid and fumarate accumulation. Other TCA cycle intermediates were not significantly changed in the *tic* mutant (Sanchez-Villarreal et al., 2013) suggesting that the phenotypes are due to an altered balance of carbon and nitrogen usage.

In mammals, the circadian regulation of metabolism relies on both systemic as well as local signals (Brown, 2016) affecting basic pathways at a cellular level and metabolic homeostasis within the whole organism (Asher and Schibler, 2011). In plants, the mitochondrial proteome varies depending on the metabolic requirements of the different tissues (des Francs-Small et al., 1992; Bardel et al., 2002; Lee et al., 2008). Based on the circadian communication between shoots and roots in *Arabidopsis* (James et al., 2008; Takahashi et al., 2015; Chen et al., 2020), it would be interesting to dissect the possible cell-, tissue-, and organ-specificity of metabolic regulation by the clock, and identify possible systemic signal(s) able to transmit energy status among different parts of the plant for improved productivity and fitness. Furthermore, metabolism-related organelles do not function in isolation within the cell but are tightly connected with other key cellular pathways including the cell cycle (Kianian and Kianian, 2014). As the circadian clock controls the timing of the cell cycle in plants (Fung-Uceda et al., 2018), it is possible that the circadian interaction with the cell cycle might also play a role regulating cellular metabolism in plants. Our results pave the way for further studies exploiting the chronobiology of metabolism to improve metabolic homeostasis and cellular energy in plants and animals.

## Data Availability Statement

The original contributions presented in the study are included in the article/Supplementary Material, further inquiries can be directed to the corresponding author.

## Author Contributions

LC-C, TY, YZ, and MO performed the experiments. AF contributed with reagents, ideas, and comments. PM conceived the project, designed the experiments, and wrote the manuscript. All authors read, revised, and approved the manuscript.

## Conflict of Interest

The authors declare that the research was conducted in the absence of any commercial or financial relationships that could be construed as a potential conflict of interest.

## Footnotes

1 http://www.leco.com/

## References

[B1] Araújo W. L. Nunes-Nesi A. Fernie A. R. (2011). Fumarate: multiple functions of a simple metabolite. *Phytochemistry* 72 838–843. 10.1016/j.phytochem.2011.02.028 21440919

[B2] Asher G. Schibler U. (2011). Crosstalk between components of circadian and metabolic cycles in mammals. *Cell Metab.* 13 125–137. 10.1016/j.cmet.2011.01.006 21284980

[B3] Atkins K. A. Dodd A. N. (2014). Circadian regulation of chloroplasts. *Curr. Opin. Plant Biol.* 21 43–50. 10.1016/j.pbi.2014.06.008 25026538

[B4] Bardel J. Louwagie M. Jaquinod M. Jourdain A. Luche S. Rabilloud T. (2002). A survey of the plant mitochondrial proteome in relation to development. *Proteomics* 2 880–898. 10.1002/1615-9861(200207)2:7<880::AID-PROT880<3.0.CO;2-0 12124934

[B5] Blasing O. E. Gibon Y. Gunther M. Hohne M. Morcuende R. Osuna D. (2005). Sugars and circadian regulation make major contributions to the global regulation of diurnal gene expression in *Arabidopsis*. *Plant Cell* 17 3257–3281. 10.1105/tpc.105.035261 16299223 PMC1315368

[B6] Brown S. A. (2016). Circadian metabolism: from mechanisms to metabolomics and medicine. *Trends Endocrinol. Metab.* 27 415–426. 10.1016/j.tem.2016.03.015 27113082

[B7] Chen W. W. Takahashi N. Hirata Y. Ronald J. Porco S. Davis S. J. (2020). A mobile ELF4 delivers circadian temperature information from shoots to roots. *Nat. Plants* 6 416–426. 10.1038/s41477-020-0634-2 32284549 PMC7197390

[B8] Chia D. W. Yoder T. J. Reiter W. D. Gibson S. I. (2000). Fumaric acid: an overlooked form of fixed carbon in *Arabidopsis* and other plant species. *Planta* 211 743–751. 10.1007/s004250000345 11089689

[B9] Chrobok D. Law S. R. Brouwer B. Lindén P. Ziolkowska A. Liebsch D. (2016). Dissecting the metabolic role of mitochondria during developmental leaf senescence. *Plant Physiol.* 172 2132–2153. 10.1104/pp.16.01463 27744300 PMC5129728

[B10] Clough S. J. Bent A. F. (1998). Floral dip: a simlified method for *Agrobacterium*-mediated transformation of *Arabidopsis thaliana*. *Plant J.* 16 735–743. 10.1046/j.1365-313X.1998.00343.x 10069079

[B11] des Francs-Small C. C. Ambard-Bretteville F. Darpas A. Sallantin M. Huet J. C. Pernollet J. C. (1992). Variation of the polypeptide composition of mitochondria isolated from different potato tissues. *Plant Physiol.* 98 273–278. 10.1104/pp.98.1.273 16668624 PMC1080179

[B12] Dibner C. Schibler U. (2015). Circadian timing of metabolism in animal models and humans. *J. Intern. Med.* 277 513–527. 10.1111/joim.12347 25599827

[B13] Dodd A. N. Salathia N. Hall A. Kevei E. Toth R. Nagy F. (2005). Plant circadian clocks increase photosynthesis, growth, survival, and competitive advantage. *Science* 309 630–633. 10.1126/science.1115581 16040710

[B14] Elhafez D. Murcha M. W. Clifton R. Soole K. L. Day D. A. Whelan J. (2006). Characterization of mitochondrial alternative NAD(P)H Dehydrogenases in *Arabidopsis*: intraorganelle location and expression. *Plant Cell Physiol.* 47 43–54. 10.1093/pcp/pci221 16258072

[B15] Fernie A. R. Aharoni A. Willmitzer L. Stitt M. Tohge T. Kopka J. (2011). Recommendations for reporting metabolite data. *Plant Cell* 23 2477–2482. 10.1105/tpc.111.086272 21771932 PMC3226225

[B16] Flis A. Mengin V. Ivakov A. A. Mugford S. T. Hubberten H. M. Encke B. (2019). Multiple circadian clock outputs regulate diel turnover of carbon and nitrogen reserves. *Plant Cell Environ.* 42 549–573. 10.1111/pce.13440 30184255

[B17] Fukushima A. Kusano M. Nakamichi N. Kobayashi M. Hayashi N. Sakakibara H. (2009). Impact of clock-associated *Arabidopsis* pseudo-response regulators in metabolic coordination. *Proc. Natl. Acad. Sci. U.S.A.* 106 7251–7256. 10.1073/pnas.0900952106 19359492 PMC2667372

[B18] Fung-Uceda J. Lee K. Seo P. J. Polyn S. De Veylder L. Mas P. (2018). The circadian clock sets the time of DNA replication licensing to regulate growth in *Arabidopsis*. *Dev. Cell* 45 101–113.e4. 10.1016/j.devcel.2018.02.022 29576425

[B19] Gendron J. M. Pruneda-Paz J. L. Doherty C. J. Gross A. M. Kang S. E. Kay S. A. (2012). *Arabidopsis* circadian clock protein, TOC1,is a DNA-binding transcription factor. *Proc. Natl. Acad. Sci. U.S.A.* 109 3167–3172. 10.1073/pnas.1200355109 22315425 PMC3286946

[B20] Gibon Y. Usadel B. Blaesing O. Kamlage B. Hoehne M. Trethewey R. (2006). Integration of metabolite with transcript and enzyme activity profiling during diurnal cycles in *Arabidopsis rosettes*. *Genome Biol.* 7:R76. 10.1186/gb-2006-7-8-R76 16916443 PMC1779593

[B21] Gill S. Le H. D. Melkani G. C. Panda S. (2015). Time-restricted feeding attenuates age-related cardiac decline in *Drosophila*. *Science* 347 1265–1269. 10.1126/science.1256682 25766238 PMC4578815

[B22] Greenham K. McClung C. R. (2015). Integrating circadian dynamics with physiological processes in plants. *Nat. Rev. Genet.* 16 598–610. 10.1038/nrg3976 26370901

[B23] Haydon M. J. Mielczarek O. Robertson F. C. Hubbard K. E. Webb A. A. R. (2013). Photosynthetic entrainment of the *Arabidopsis thaliana* circadian clock. *Nature* 502 689–692. 10.1038/nature12603 24153186 PMC3827739

[B24] Hsu P. Y. Devisetty U. K. Harmer S. L. (2013). Accurate timekeeping is controlled by a cycling activator in *Arabidopsis*. *eLife* 2013:e00473. 10.7554/eLife.00473 23638299 PMC3639509

[B25] Huang W. Pérez-García P. Pokhilko A. Millar A. J. Antoshechkin I. Riechmann J. L. (2012). Mapping the core of the *Arabidopsis* circadian clock defines the network structure of the oscillator. *Science* 336 75–79. 10.1126/science.1219075 22403178

[B26] Ishizaki K. Larson T. R. Schauer N. Fernie A. R. Graham I. A. Leaver C. J. (2005). The critical role of *Arabidopsis* electron-transfer flavoprotein:ubiquinone oxidoreductase during dark-induced starvation. *Plant Cell* 17 2587–2600. 10.1105/tpc.105.035162 16055629 PMC1197437

[B27] Ishizaki K. Schauer N. Larson T. R. Graham I. A. Fernie A. R. Leaver C. J. (2006). The mitochondrial electron transfer flavoprotein complex is essential for survival of *Arabidopsis* in extended darkness. *Plant J.* 47 751–760. 10.1111/j.1365-313X.2006.02826.x 16923016

[B28] James A. B. Monreal J. A. Nimmo G. A. Kelly C. L. Herzyk P. Jenkins G. I. (2008). The circadian clock in *Arabidopsis* roots is a simplified slave version of the clock in shoots. *Science* 322 1832–1835. 10.1126/science.1161403 19095940

[B29] Kianian P. M. A. Kianian S. F. (2014). Mitochondrial dynamics and the cell cycle. *Front. Plant Sci.* 5:222. 10.3389/fpls.2014.00222 24904617 PMC4035010

[B30] Kim H. Kim Y. Yeom M. Lim J. Nam H. G. (2016). Age-associated circadian period changes in *Arabidopsis* leaves. *J. Exp. Bot.* 67 2665–2673. 10.1093/jxb/erw097 27012281 PMC4861015

[B31] Kopka J. Schauer N. Krueger S. Birkemeyer C. Usadel B. Bergmüller E. (2005). GMD@CSB.DB: the Golm metabolome database. *Bioinformatics* 21 1635–1638. 10.1093/bioinformatics/bti236 15613389

[B32] Lee C. P. Eubel H. Millar A. H. (2010). Diurnal changes in mitochondrial function reveal daily optimization of light and dark respiratory metabolism in *Arabidopsis*. *Mol. Cell. Proteomics* 9 2125–2139. 10.1074/mcp.M110.001214 20601493 PMC2953910

[B33] Lee C. P. Eubel H. O’Toole N. Millar A. H. (2008). Heterogeneity of the mitochondrial proteome for photosynthetic and non-photosynthetic *Arabidopsis* metabolism. *Mol. Cell. Proteomics* 7 1297–1316. 10.1074/mcp.M700535-MCP200 18385124

[B34] Lim S. L. Voon C. P. Guan X. Yang Y. Gardeström P. Lim B. L. (2020). In planta study of photosynthesis and photorespiration using NADPH and NADH/NAD+ fluorescent protein sensors. *Nat. Commun.* 11 1–12. 10.1038/s41467-020-17056-0 32591540 PMC7320160

[B35] Lisec J. Schauer N. Kopka J. Willmitzer L. Fernie A. R. (2006). Gas chromatography mass spectrometry–based metabolite profiling in plants. *Nat. Protoc.* 1 387–396. 10.1038/nprot.2006.59 17406261

[B36] Livak K. J. Schmittgen T. D. (2001). Analysis of relative gene expression data using real-time quantitative PCR and the 2−ΔΔCT Method. *Methods* 25 402–408. 10.1006/meth.2001.1262 11846609

[B37] Luedemann A. von Malotky L. Erban A. Kopka J. (2012). “TagFinder: preprocessing software for the fingerprinting and the profiling of gas chromatography–mass spectrometry based metabolome analyses,” in *Plant Metabolomics: Methods and Protocols*, eds Hardy N. W. Hall R. D. (Totowa, NJ: Humana Press), 255–286. 10.1007/978-1-61779-594-7_16 22351182

[B38] Ma Y. Gil S. Grasser K. D. Mas P. (2018). Targeted recruitment of the basal transcriptional machinery by LNK clock components controls the circadian rhythms of nascent RNAs in *Arabidopsis*. *Plant Cell* 30 907–924. 10.1105/tpc.18.00052 29618629 PMC5973845

[B39] Makino S. Matsushika A. Kojima M. Yamashino T. Mizuno T. (2002). The APRR1/TOC1 quintet implicated in circadian rhythms of *Arabidopsis thaliana*: characterization with APRR1-overexpressing plants. *Plant Cell Physiol.* 43 58–69. 10.1093/pcp/pcf005 11828023

[B40] Más P. Alabadí D. Yanovsky M. J. Oyama T. Kay S. A. (2003a). Dual role of TOC1 in the control of circadian and photomorphogenic responses in *Arabidopsis*. *Plant Cell* 15 223–236. 10.1105/tpc.006734 12509533 PMC143493

[B41] Más P. Kim W.-Y. Somers D. E. Kay S. A. (2003b). Targeted degradation of TOC1 by ZTL modulates circadian function in *Arabidopsis thaliana*. *Nature* 426 567–570. 10.1038/nature02163 14654842

[B42] McClung C. R. Hsu M. Painter J. E. Gagne J. M. Karlsberg S. D. Salomé P. A. (2000). Integrated temporal regulation of the photorespiratory pathway. circadian regulation of two *Arabidopsis* Genes encoding serine hydroxymethyltransferase. *Plant Physiol.* 123 381–392. 10.1104/pp.123.1.381 10806255 PMC59012

[B43] Michael T. P. Mockler T. C. Breton G. McEntee C. Byer A. Trout J. D. (2008). Network discovery pipeline elucidates conserved time-of-day-specific cis-regulatory modules. *PLoS Genet.* 4:e14. 10.1371/journal.pgen.0040014 18248097 PMC2222925

[B44] Michalecka A. M. Svensson ÅS. Johansson F. I Agius S. C. Johanson U. Brennicke A. (2003). *Arabidopsis* genes encoding mitochondrial Type II NAD(P)H Dehydrogenases have different evolutionary origin and show distinct responses to light. *Plant Physiol* 133 642–652. 10.1104/pp.103.024208 12972666 PMC219040

[B45] Millar A. H. Whelan J. Soole K. L. Day D. A. (2011). Organization and regulation of mitochondrial respiration in plants. *Annu. Rev. Plant Biol.* 62 79–104. 10.1146/annurev-arplant-042110-103857 21332361

[B46] Mockler T. C. Michael T. P. Priest H. D. Shen R. Sullivan C. M. Givan S. A. (2007). The DIURNAL project: DIURNAL and circadian expression profiling, model-based pattern matching, and promoter analysis. *Cold Spring Harb. Symp. Quant. Biol.* 72 353–363. 18419293 10.1101/sqb.2007.72.006

[B47] Nakagawa T. Kurose T. Hino T. Tanaka K. Kawamukai M. Niwa Y. (2007a). Development of series of gateway binary vectors, pGWBs, for realizing efficient construction of fusion genes for plant transformation. *J. Biosci. Bioeng.* 104 34–41. 10.1263/jbb.104.34 17697981

[B48] Nakagawa T. Suzuki T. Murata S. Nakamura S. Hino T. Maeo K. (2007b). Improved gateway binary vectors: high-performance vectors for creation of fusion constructs in transgenic analysis of plants. *Biosci. Biotechnol. Biochem.* 71 2095–2100. 10.1271/bbb.70216 17690442

[B49] Niwa Y. Ito S. Nakamichi N. Mizoguchi T. Niinuma K. Yamashino T. (2007). Genetic linkages of the circadian clock-associated genes, TOC1, CCA1 and LHY, in the photoperiodic control of flowering time in *Arabidopsis thaliana*. *Plant Cell Physiol.* 48 925–937. 10.1093/pcp/pcm067 17540692

[B50] Noordally Z. B. Ishii K. Atkins K. A. Wetherill S. J. Kusakina J. Walton E. J. (2013). Circadian control of chloroplast transcription by a nuclear-encoded timing signal. *Science* 339 1316–1319. 10.1126/science.1230397 23493713

[B51] Nunes-Nesi A. Araújo W. L. Fernie A. R. (2011). Targeting mitochondrial metabolism and machinery as a means to enhance photosynthesis. *Plant Physiol.* 155 101–107. 10.1104/pp.110.163816 20966153 PMC3075771

[B52] Nunes-Nesi A. Araújo W. L. Obata T. Fernie A. R. (2013). Regulation of the mitochondrial tricarboxylic acid cycle. *Curr. Opin. Plant Biol.* 16 335–343. 10.1016/j.pbi.2013.01.004 23462640

[B53] Nunes-Nesi A. Carrari F. Gibon Y. Sulpice R. Lytovchenko A. Fisahn J. (2007). Deficiency of mitochondrial fumarase activity in tomato plants impairs photosynthesis via an effect on stomatal function. *Plant J.* 50 1093–1106. 10.1111/j.1365-313X.2007.03115.x 17461782

[B54] Pedrotti L. Weiste C. Nägele T. Wolf E. Lorenzin F. Dietrich K. (2018). Snf1-RELATED KINASE1-controlled C/S1-bZIP signaling activates alternative mitochondrial metabolic pathways to ensure plant survival in extended darkness. *Plant Cell* 30 495–509. 10.1105/tpc.17.00414 29348240 PMC5868691

[B55] Perales M. Más P. (2007). A functional link between rhythmic changes in chromatin structure and the *Arabidopsis* biological clock. *Plant Cell* 19 2111–2123. 10.1105/tpc.107.050807 17616736 PMC1955692

[B56] Pokhilko A. Fernandez A. P. Edwards K. D. Southern M. M. Halliday K. J. Millar A. J. (2012). The clock gene circuit in *Arabidopsis* includes a repressilator with additional feedback loops. *Mol. Syst. Biol.* 8:574. 10.1038/msb.2012.6 22395476 PMC3321525

[B57] Pracharoenwattana I. Zhou W. Keech O. Francisco P. B. Udomchalothorn T. Tschoep H. (2010). *Arabidopsis* has a cytosolic fumarase required for the massive allocation of photosynthate into fumaric acid and for rapid plant growth on high nitrogen. *Plant J.* 62 785–795. 10.1111/j.1365-313X.2010.04189.x 20202172

[B58] Sanchez S. E. Kay S. A. (2016). The Plant circadian clock: from a simple timekeeper to a complex developmental manager. *Cold Spring Harb. Perspect. Biol.* 8:a027748. 10.1101/cshperspect.a027748 27663772 PMC5131769

[B59] Sanchez-Villarreal A. Shin J. Bujdoso N. Obata T. Neumann U. Du S.-X. (2013). TIME FOR COFFEE is an essential component in the maintenance of metabolic homeostasis in *Arabidopsis thaliana*. *Plant J.* 76 188–200. 10.1111/tpj.12292 23869666

[B60] Schauer N. Steinhauser D. Strelkov S. Schomburg D. Allison G. Moritz T. (2005). GC-MS libraries for the rapid identification of metabolites in complex biological samples. *FEBS Lett.* 579 1332–1337. 10.1016/j.febslet.2005.01.029 15733837

[B61] Schneider C. A. Rasband W. S. Eliceiri K. W. (2012). NIH Image to ImageJ: 25 years of image analysis. *Nat. Methods* 9 671–675. 10.1038/nmeth.2089 22930834 PMC5554542

[B62] Smith A. M. Stitt M. (2007). Coordination of carbon supply and plant growth. *Plant Cell Environ.* 30 1126–1149. 10.1111/j.1365-3040.2007.01708.x 17661751

[B63] Somers D. E. Webb A. A. R. Pearson M. Kay S. A. (1998). The short-period mutant toc1-1, alters circadian clock regulation of multiple outputs throughout development in *Arabidopsis thaliana*. *Development* 125 485–494. 9425143 10.1242/dev.125.3.485

[B64] Strayer C. Oyama T. Schultz T. F. Raman R. Somers D. E. Mas P. (2000). Cloning of the *Arabidopsis* clock gene TOC1, an autoregulatory response regulator homolog. *Science* 289 768–771. 10.1126/science.289.5480.768 10926537

[B65] Sweetlove L. J. Fernie A. R. (2013). The spatial organization of metabolism within the plant cell. *Annu. Rev. Plant Biol.* 64 723–746. 10.1146/annurev-arplant-050312-120233 23330793

[B66] Takahashi N. Hirata Y. Aihara K. Mas P. (2015). A hierarchical multi-oscillator network orchestrates the *Arabidopsis* circadian system. *Cell* 163 148–159. 10.1016/j.cell.2015.08.062 26406375

[B67] Taylor N. L. Heazlewood J. L. Day D. A. Millar A. H. (2004). Lipoic acid-dependent oxidative catabolism of α-keto acids in mitochondria provides evidence for branched-chain amino acid catabolism in *Arabidopsis*. *Plant Physiol.* 134 838–848. 10.1104/pp.103.035675 14764908 PMC344558

[B68] Tschoep H. Gibon Y. Carillo P. Armengaud P. Szecowka M. Nunes-Nesi A. (2009). Adjustment of growth and central metabolism to a mild but sustained nitrogen-limitation in *Arabidopsis*. *Plant Cell Environ.* 32 300–318. 10.1111/j.1365-3040.2008.01921.x 19054347

[B69] Xing H. Wang P. Cui X. Zhang C. Wang L. Liu X. (2015). LNK1 and LNK2 recruitment to the evening element require morning expressed circadian related MYB-like transcription factors. *Plant Signal. Behav.* 10:e1010888. 10.1080/15592324.2015.1010888 25848708 PMC4622603

[B70] Xu K. Zheng X. Sehgal A. (2008). Regulation of feeding and metabolism by neuronal and peripheral clocks in *Drosophila*. *Cell Metab.* 8 289–300. 10.1016/j.cmet.2008.09.006 18840359 PMC2703740

[B71] Yamashino T. Ito S. Niwa Y. Kunihiro A. Nakamichi N. Mizuno T. (2008). Involvement of *Arabidopsis* clock-associated pseudo-response regulators in diurnal oscillations of gene expression in the presence of environmental time cues. *Plant Cell Physiol.* 49 1839–1850. 10.1093/pcp/pcn165 19015137

[B72] Zhang Y. Krahnert I. Bolze A. Gibon Y. Fernie A. R. (2020). Adenine nucleotide and nicotinamide adenine dinucleotide measurements in plants. *Curr. Protoc. Plant Biol.* 5:e20115. 10.1002/cppb.20115 32841544

