# Supplementary material for: Circadian Control of Metabolism by the Clock Component TOC1
Source: Front Plant Sci. 2021 Jun 14;12:683516. doi: 10.3389/fpls.2021.683516 (PMC8238050; doi:10.3389/fpls.2021.683516)
Supplement: Supplementary file 2 [file Data_Sheet_1.pdf]

Supplementary Material

Supplementary Figures

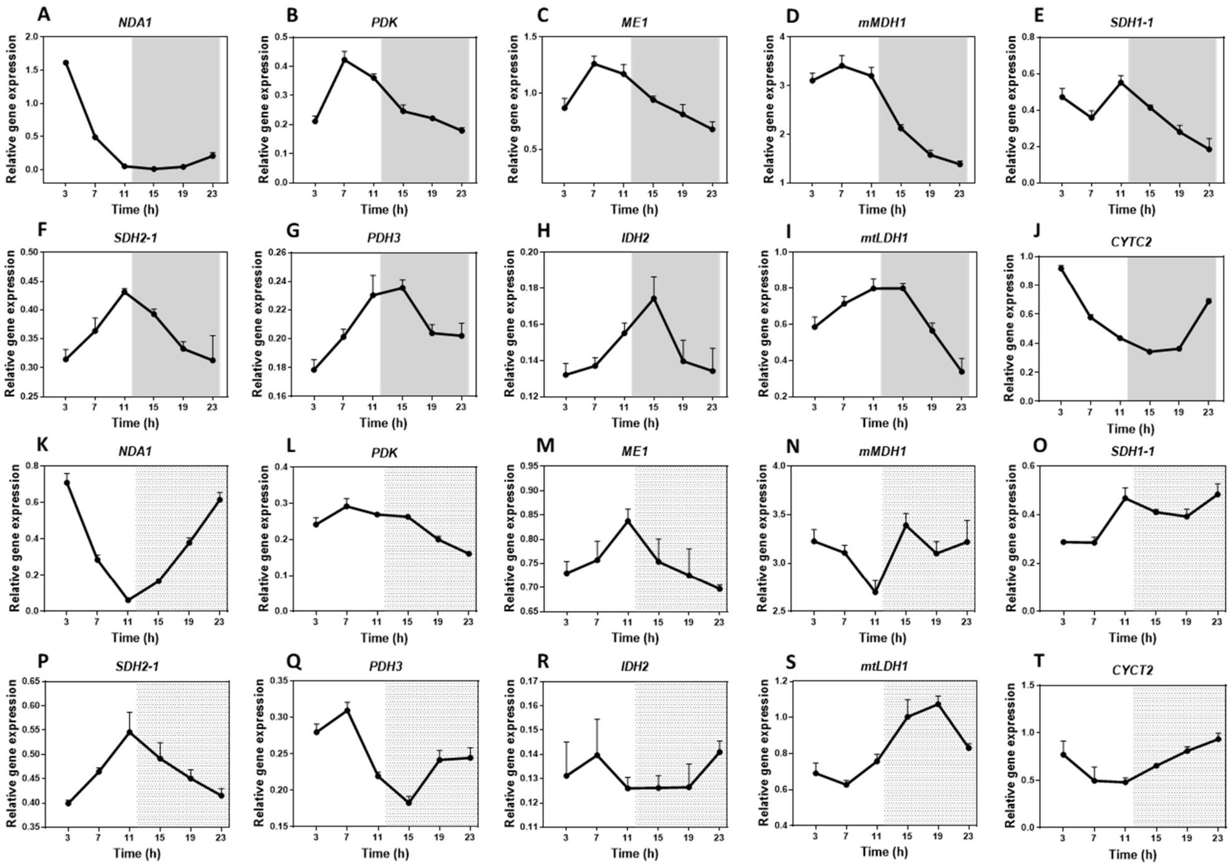

Supplementary Figure 1. Diurnal and circadian expression of mitochondrial-related genes. Time course analyses of a subset of mitochondrial-related genes from seedlings grown under (A-J) 12h light: 12h dark cycles or (K-T) after transferring to constant light conditions (LL). Samples were collected and analyzed every four hours over a diurnal or circadian cycle. Data is represented as the mean + SEM. The white and gray areas in (A-J) represent day and night, respectively. The white and dotted areas in (K-T) represent subjective day and subjective night, respectively. Two biological replicates were performed for all experiments, with plants grown independently, with samples collected, processed and analyzed at different times. *ALTERNATIVE NAD(P)H DEHYDROGENASE 1 (NDA1), PYRUVATE DEHYDROGENASE KINASE (PDK), NAD-DEPENDENT MALIC ENZYME 1 (ME1), MITOCHONDRIAL MALATE DEHYDROGENASE 1 (mMDH1), SUCCINATE DEHYDROGENASE 2-1 (SDH2-1), MITOCHONDRIAL PYRUVATE DEHYDROGENASE SUBUNIT 2-1 (PDH3), ISOCITRATE DEHYDROGENASE SUBUNIT 2 (IDH2), LIPOAMIDE DEHYDROGENASE 1 (mtLDH1), CYTOCHROME C-2 (CYCT2).*

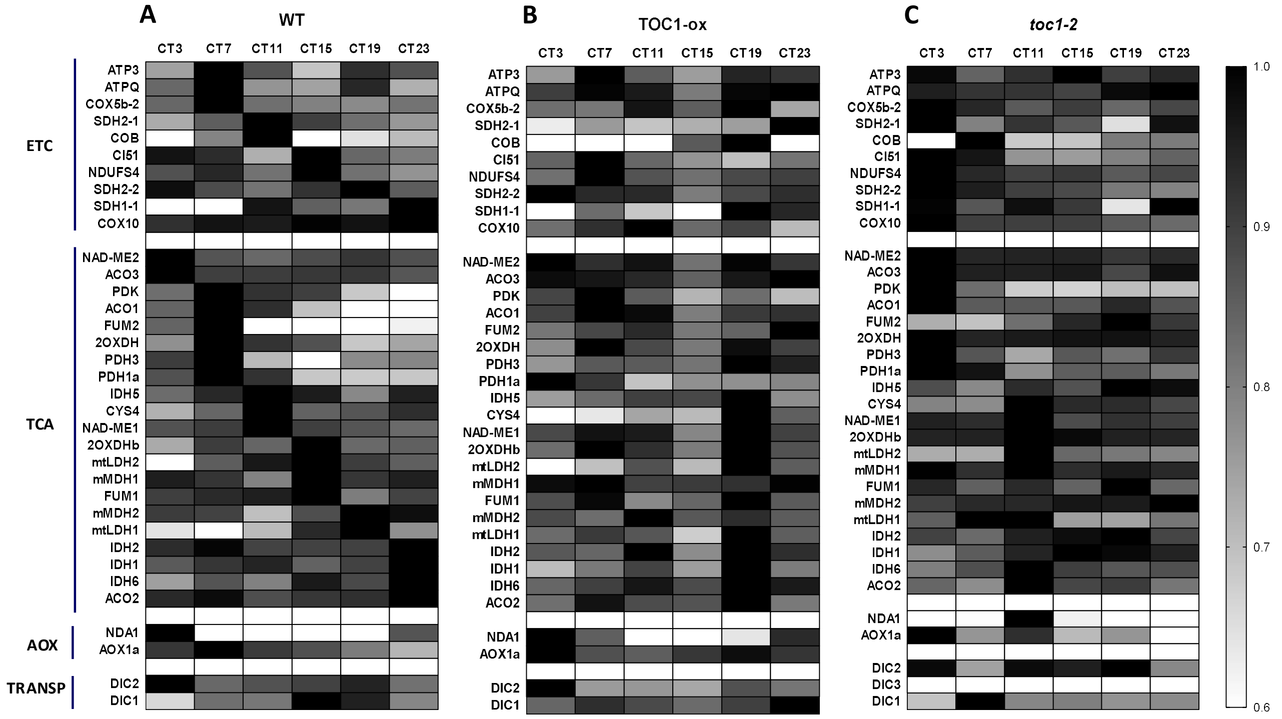

Supplementary Figure 2. TOC1 regulates the circadian expression of mitochondrial-related genes. Heat map showing the time course analyses of mitochondrial-related gene expression over a circadian cycle under constant light (LL) conditions. (A) WT, (B) TOC1-ox and (C) *toc1-2* plants were entrained under 12h light: 12h dark cycles and transferred for 2 days to LL. The expression of selected genes encoding proteins belonging to the mitochondrial electron transport chain (ETC), the tricarboxylic acid (TCA) cycle, NADH dehydrogenases, alternative oxidases (AOX) as well as mitochondrial transporters (TRANSP) was analyzed. Individual waveforms are shown in Figure 2. The average gene expression values at every time point was normalized to the peak value for each gene. Relative expression was obtained by RT Q-PCR analyses.

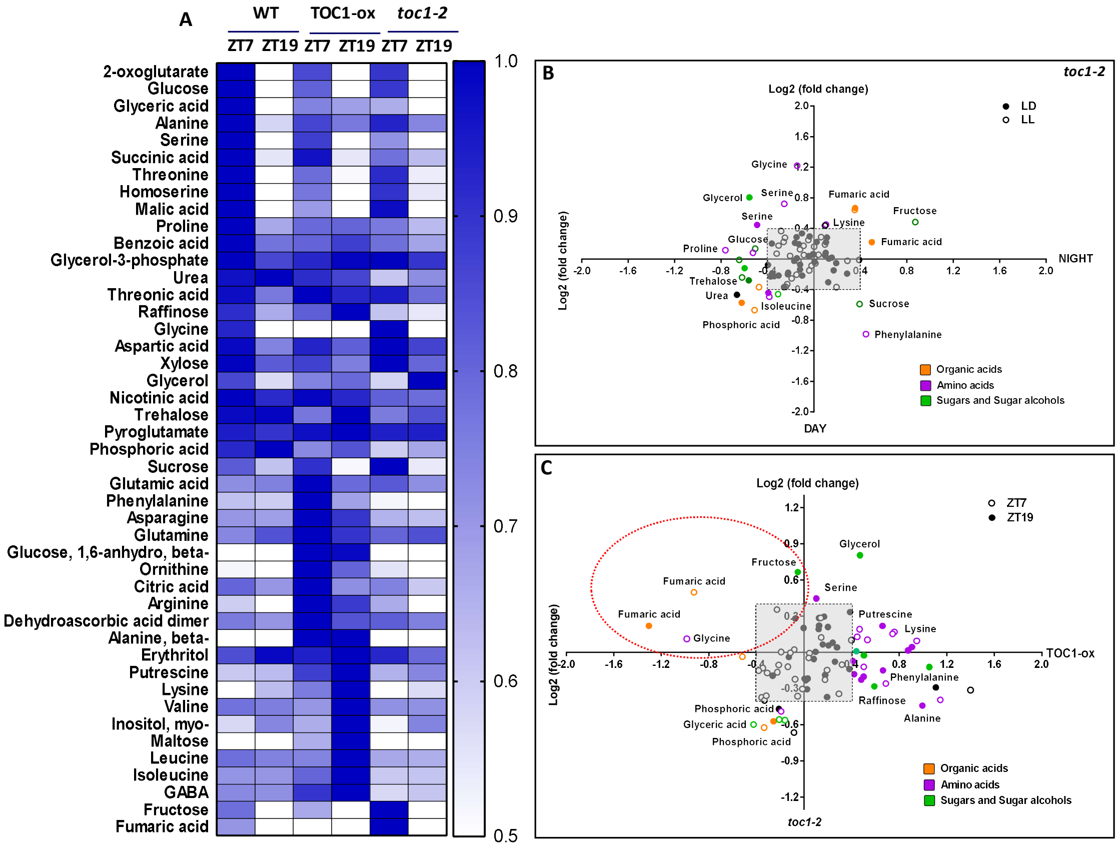

Supplementary Figure 3. TOC1 regulates the diurnal and circadian accumulation of metabolites. (A) Heat map of GC-MS-analyzed metabolites in WT, TOC1-ox and *toc1-2* plants. Plants were entrained under 12h light: 12h dark cycles before sampling at ZT7 and ZT19. (B) Dispersion plot of metabolite fold change (day versus night) under LD (12h light: 12h dark cycles) and LL (subjective day versus subjective night) in *toc1-2* relative to WT. (C) Dispersion plot of metabolite fold change (*toc1-2* versus TOC1-ox) under 12h light: 12h dark cycles. Metabolites were clustered per class into organic acids (orange), amino acids (purple), and sugars and sugar alcohols (green). Relative metabolite content was normalized to the mean of WT plants and fold-change values were log2 transformed. Log2 fold differences < [0.4] were not considered (pale gray square). Orange dotted oval denotes metabolites down-regulated in TOC1-ox and up-regulated in *toc1-2*. Data are the result of 5 biological replicates, with plants grown independently.

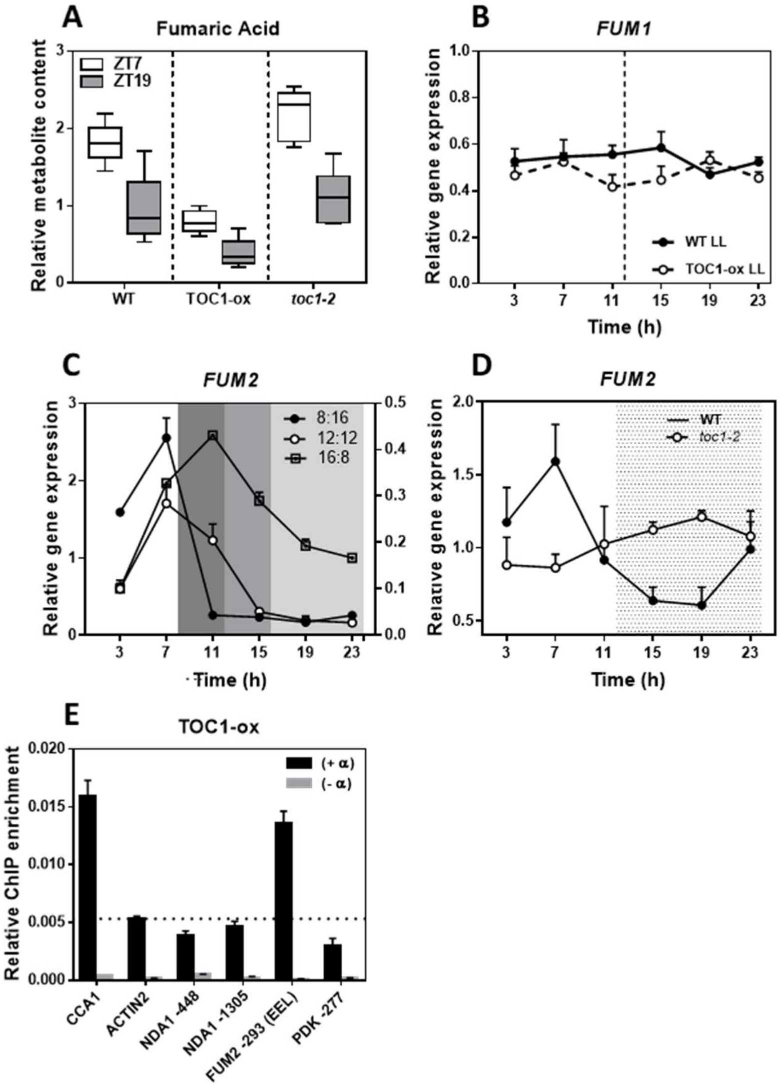

Supplementary Figure 4. TOC1 controls the diurnal and circadian expression of *FUM2* by direct binding to *FUM2* gene promoter. Relative fumaric acid content in WT, TOC1-ox and *toc1-2* plants entrained under 12h light: 12h dark cycles and sampled at ZT7 and ZT19. Five biological replicates were performed with plants grown independently, with samples collected, processed and analyzed at different times. (B) Time course analyses of *FUM1* expression in WT and TOC1-ox plants grown under 12h light: 12h dark cycles and transferred for 2 days to constant light (LL) before sampling. (C) Time course analyses of *FUM2* expression in WT plants grown at the indicated photoperiods. Right Y -axis shows relative gene expression in 16:8 photoperiod. (D) Time course analyses of *FUM2* expression in WT and *toc1-2* plants grown under 12h light: 12h dark cycles transferred for 2 days to constant light (LL) before sampling. (E) Chromatin immunoprecipitation (ChIP) assays with TOC1-ox plants grown under 12h light: 12h dark cycles and sampled at ZT7. ChIP enrichment was calculated relative to the input. Samples were incubated with an anti-MYC antibody (+α) or without antibody (-α). Two biological replicates (B, C) or three (D, E) were performed for the experiments, with plants grown independently, with samples collected, processed and analyzed at different times. Values are means + SEM. The white and gray areas in (C) represent day and night, respectively. The white and dotted areas in (D) represent subjective day and subjective night, respectively.

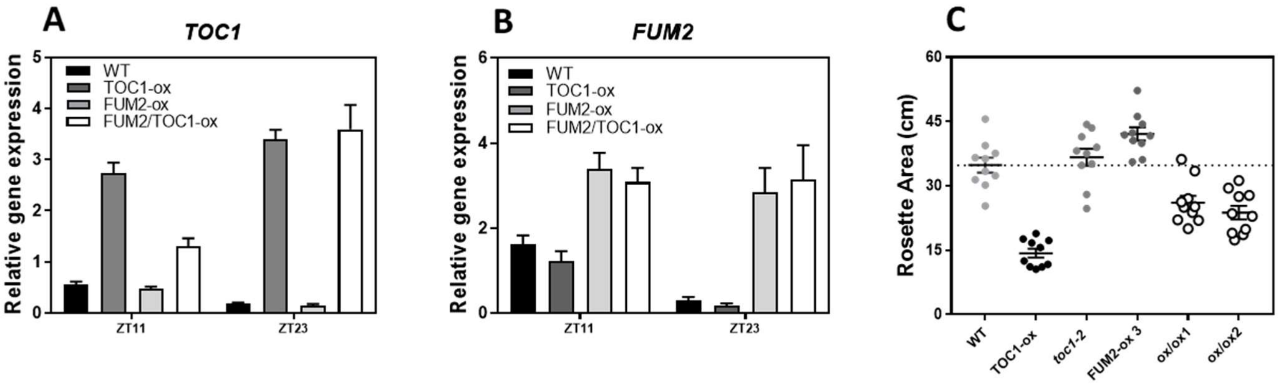

Supplementary Figure 5. Genetic interaction analyses of TOC1 and FUM2. (A) *TOC1* and (B) *FUM2* gene expression analyses by RT-QPCR at ZT11 and ZT23 in plants grown under LD cycles. Data are represented as the mean + SEM. (C) Rosette area of the indicated genotypes grown under long day (LgD) conditions. Every dot represents a single rosette. Dotted line shows the mean of WT. Three biological replicates were performed for all experiments, with plants grown independently, with samples collected, processed and analyzed at different times.

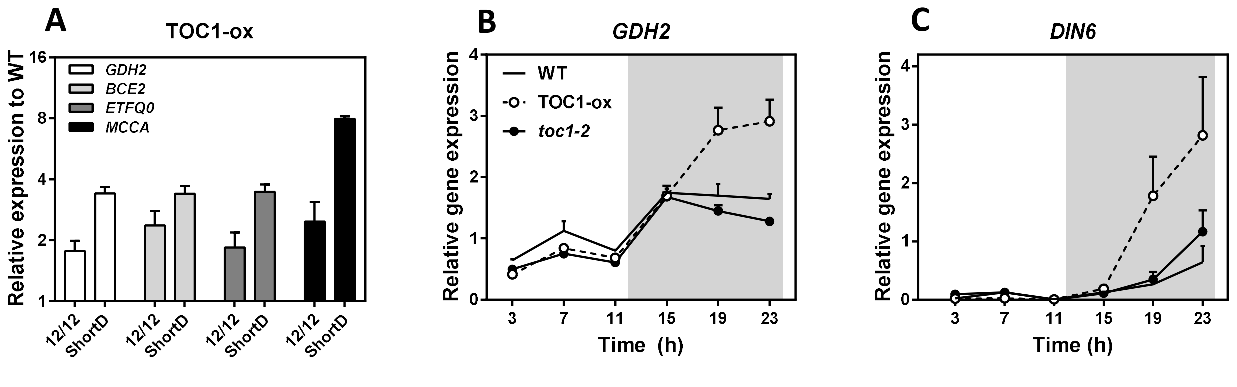

Supplementary Figure 6. Genetic interaction analyses of TOC1 and FUM2. (A) *GDH2*, *BCE2*, *ETFQO* and *MCCA* gene expression analyses by RT-QPCR at ZT23 in plants grown under LD cycles. Time course analysis by RT-QPCR of (B) *GDH2* and (C) *DIN6* gene expression in the indicated genotypes grown under LD cycles. Data are represented as the mean + SEM. The white and gray areas represent day and night, respectively. Three biological replicates were performed for all experiments, with plants grown independently, with samples collected, processed and analyzed at different times.
